# Long term sequelae after SARS-CoV-2 infection in children: a household study

**DOI:** 10.1186/s12985-023-02094-z

**Published:** 2023-06-28

**Authors:** Judith G. C. Sluiter-Post, Elandri Fourie, Joanne G. Wildenbeest, Steven F. L. van Lelyveld, Patricia C. J. L. Bruijning-Verhagen, Marianne A. van Houten

**Affiliations:** 1 Spaarne Gasthuis Academy, Hoofddorp and Haarlem, The Netherlands; 2 Department of Paediatric Infectious Diseases and Immunology, Wilhelmina Children’s Hospital University Medical Center Utrecht, Utrecht, The Netherlands; 3 Department of Internal Medicine, Spaarne Gasthuis, Hoofddorp and Haarlem, The Netherlands; 4 Department of Epidemiology, Julius Centre for Health Sciences and Primary Care, University Medical Centre Utrecht, Utrecht, The Netherlands; 5 Department of Paediatrics, Spaarne Gasthuis, Hoofddorp and Haarlem, Spaarnepoort 1, 2134 TM Hoofddorp, The Netherlands

**Keywords:** Long term symptoms, SARS-CoV-2, Children, Household

## Abstract

**Background:**

In children persistent symptoms after SARS-CoV-2 infection have been reported, however, duration and characteristics of symptoms in previously healthy children remain unclear. Therefore this study aimed to evaluate persisting symptoms in children at 6 and 12 months after a SARS-CoV-2 infection.

**Methods:**

In this prospective cohort study households with a confirmed SARS-CoV-2 positive outbreak were matched 1:1 to household controls from SARS-CoV-2 negative outbreaks. These households completed questionnaires at 6 and 12 months on the presence and severity of SARS-CoV-2 related symptoms, general well-being/functioning, cognition, persisting symptoms and quality of life.

**Findings:**

None of the children who had a SARS-CoV-2 infection during the study reported persistent symptoms at 6 and 12 months after infection, whereas almost 8% of the children with a negative RT-PCR test during the study reported symptoms such as coughing and mild fever, however, no significant differences were found. In addition, for all other outcomes, no differences were observed between the two groups.

**Take home message:**

Post-acute sequelae of mild SARS-CoV-2 infections appears to be uncommon in previously healthy children.

**Supplementary Information:**

The online version contains supplementary material available at 10.1186/s12985-023-02094-z.

## Introduction

The persistence of long-term symptoms after a SARS-CoV-2 infection have been reported in several studies. However, data on long-term sequela of SARS-CoV-2 in children, especially after mild or asymptomatic infections, are limited. Until now symptoms such as fatigue, muscle and joint pain, headache and respiratory problems are reported. This is in line with symptoms described in adults [1–3].

Accurate knowledge about the percentage of children with persisting symptoms after a mild or asymptomatic SARS-CoV-2 infection is needed. Especially when incidence is high, treatment guidelines such as adjustments to vaccination programs and control precautions, such as self-isolation, would be desired. Furthermore, when persisting symptoms are common among children, education and recognition of complaints as well as management of expectations of the duration of recovery are essential.

Therefore, the aim of this study was to evaluate persisting symptoms at 6 and 12 months after (SARS-CoV-2) infections in a subset of households participating in the CoKids study [4].

## Methods

**Fig. 1:**
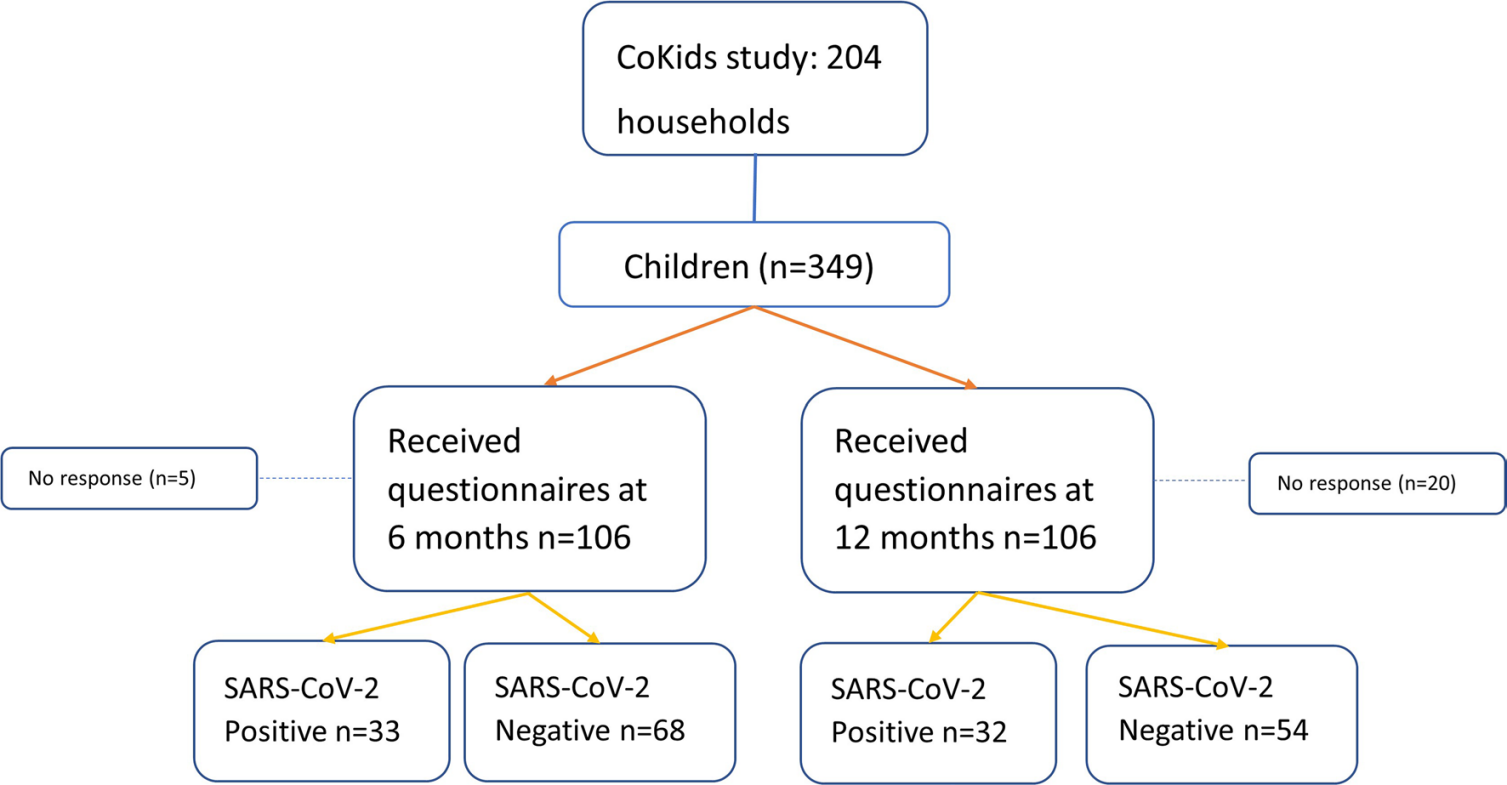
Flowchart of questionnaire response between child participants from SARS-CoV-2 positive and negative households

The subset of 204 households participating in the CoKids [4] study consisted of 459 adults and 349 children who were recruited from the Spaarne Gasthuis (The Netherlands) and prospectively followed between August 2020 and July 2021, covering the second and third wave of the COVID-19 pandemic in the Netherlands (Fig. 1). Vaccination of children had not yet started. Upon onset of respiratory symptoms in any of the household members, a household outbreak phase was initiated and lasted a minimum of 21 days. All household members were tested for SARS-CoV-2 using a combined oropharyngeal and mid-turbinate nasal swab, which was then analysed within 48 h by reverse transcriptase polymerase chain reaction (RT-PCR). Whenever a next household member developed respiratory symptoms, that individual was promptly tested again using RT-PCR. Household outbreaks of respiratory illness were divided and classified as either SARS-CoV-2 positive or negative based on the outcome of the RT-PCR. Symptoms such as fatigue, loss of smell/taste, muscle aches, runny nose, cold shivers, dyspnea, fever, sore throat, headaches, cough, diarrhoea and vomiting were logged on a daily basis for all household members during the outbreak. Data on severity of symptoms were collected using a 5-point investigator-designed scale, with higher severity scores indicating greater symptom severity [4].

Not all household members reported symptoms or experienced illness during an outbreak.

All households in a confirmed SARS-CoV-2 positive outbreak were matched 1:1 to household controls from SARS-CoV-2 negative outbreaks. Matching was based on timing of the outbreak and household composition (ages and household size). These households completed questionnaires at 6 and 12 months after a household outbreak on the presence and severity of SARS-CoV-2 related symptoms, general well-being/functioning, cognition, persisting symptoms and Quality of Life (QoL), resulting in 30.4% (106/349) of the children receiving a questionnaire. The generic QoL tools used in this study are the TNO-AZL Preschool Children's Quality of Life questionnaire (TAPQOL) questionnaire for children 0–2 years old (proxy report) and the Paediatric Quality of Life Inventory (PedSQL) questionnaire in all children aged 2–18 years old (proxy report for children < 8 years old). The PedsQL and TAPQOL questionnaires measure Health Related Quality of Life are well translated, validated questionnaires [5–8]. The TAPQOL questionnaire is used to evaluate the impact of disease on the lives of young children (from 6 months of age) in different domains (physical, social and psychological). Questions such as “Did your child sleep restlessly?” or “Did your child have a poor appetite?” could be answered with 0 “Not at all”, 1 “Rarely”, 2 “Sometimes” or 3 “Often” by parents or caregivers. The PedsQL questionnaire has four domains (Physical, Emotional, social and school functioning). Questions like “In the past week, how often has your child had problems with: playing, sleeping, running” could be answered with 0 “Not at all”, 1 “Rarely”, 2 “Sometimes”, 3 “Often” or 4 “almost always” by parents or caregivers (see Additional pdf files 1, 2, 3, 4 for the corresponding questionnaires used).

We compared presence of (persistent) clinical symptoms, general well-being/functioning, cognition, and Quality of Life (QoL) at 6 and 12 months between children in a SARS-CoV-2 positive versus SARS-CoV-2 negative respiratory illness household outbreak.

Medians alongside interquartile range (IQR) were computed for skewed distributed variables that are continuous and medians alongside standard deviation (SD) were computed for continuous variables that are normally distributed. Categorical variables were described as numbers with their corresponding percentages.

Furthermore, we calculated the long term symptom rates, as reported in the 6 months and 12 months questionnaires, using the chi-square test and described the variety of symptoms in all the different domains.

Two-tailed *P* < 0.05 was considered statistically significant. Statistical analyses were performed using R software, version 4.0.3 (R Foundation for Statistical Computing).

## Results

In total 106 of the 349 participating children in the CoKids study received questionnaires of whom 101 (95.3%) completed the 6 months questionnaires and 86 (81.0%) the 12 months questionnaires. Of the 101 children with at least one completed questionnaire, 33 were from SARS-CoV-2 positive and 68 from matched SARS-CoV-2 negative outbreaks.

No significant differences were found for household size, age distribution and gender between the households with and those without a SARS-CoV-2 infection (Table 1). In our population disease burden of acute SARS-CoV-2 infections in all child participants was mild and similar to other common respiratory illnesses.

**Table 1:** Characteristics

	SARS-CoV-2 negative	SARS-CoV-2 positive	*p* value
N = 101*	68	33	
Age (median [IQR])	4.00 [3.00, 7.00]	3.00 [3.00, 7.00]	0.622
Sex = female (%)	36 (52.9%)	15 (45.5%)	0.622
Household size (median [IQR])	4.00 [4.00, 5.00]	4.00 [4.00, 5.00]	0.400
Chronic diseases	2 (2.9%)	0 (0.0%)	0.815
BMI**			0.623
Underweight	4 (5.9%)	0 (0.0%)	
Normal weight	43 (63.2%)	22 (66.7%)	
Overweight	8 (11.8%)	4 (12.1%)	
Obesity	1 (1.5%)	0 (0.0%)	

*Number of children that completed the 6 month questionnaires

**Children under 2 years of age excluded (n = 12 SARS-CoV-2 negative group, n = 7 SARS-CoV-2 positive group)

**Fig. 2:**
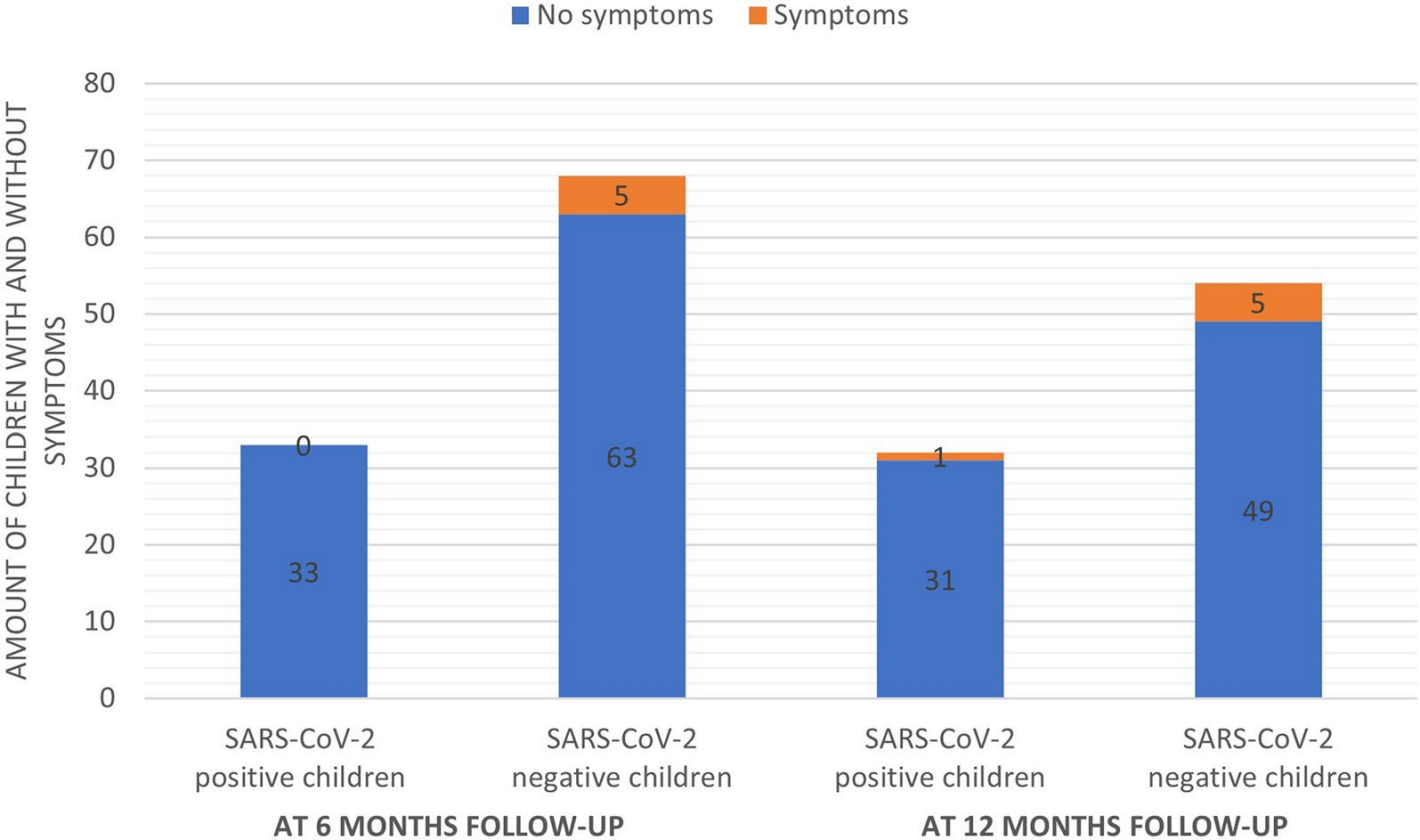
Incidence of persistent symptoms after (SARS-CoV-2) infection in children

The incidence of persisting symptoms 6 months after an infection in children with a positive SARS-CoV-2 infection was 0% (0/33) and those with a SARS-CoV2 negative respiratory illness was 7.4% (5/68). Reported symptoms were coughing and mild fever. At 12 months, the incidence of persisting symptoms was 3.1% (1/32) and 9.3% (5/54) in the SARS-CoV-2 positive and SARS-CoV-2 negative group respectively (Fig. 2). One participant in the SARS-CoV-2 positive group reported fatigue and five participants in the SARS-CoV-2 negative group reported among others symptoms such as coughing, mild fever and headache. None of the participants who reported symptoms at 12 months had expressed symptoms at 6 months after infection. For all other outcomes, such as general well-being/functioning, cognition and quality of life at 6 and 12 months, no differences were observed between the two groups.

## Discussion

In our group of 33 unvaccinated, previously healthy children, SARS-CoV-2 infections did not lead to persistent symptoms up to 6 months and 12 months after the initial infection. This is in line with data from previous studies showing that long-term symptoms after SARS-CoV-2 infections are uncommon in healthy children [9–11]. No differences were found regarding long-term symptoms in children with SARS-CoV-2 infections versus children with a negative SARS-CoV-2 RT-PCR test.

Some studies do report persisting SARS-CoV-2 symptoms in children, however, these studies have a shorter follow-up time and a higher median age of children (mean/median age 11–13 years) [1, 3, 11].

Atchison et al. evaluated the prevalence of persisting symptoms after 3 months in children aged 5–17 years. They found a prevalence of 4.4% for one or more reported persistent symptoms in children aged 5–11 years. In addition, they reported older children (> 12 years of age) had a 3 times higher chance to suffer from persistent complaints 3 months after infection [12]. Furthermore, another study, including children aged 0–17 years, reported that persistent symptoms in SARS-CoV-2 positive children of 6–17 years old were more frequent than in the control group as well as ‘long COVID’ symptoms being resolved within 1–5 months [13].

This might suggest that persistent symptoms after SARS-CoV-2 infection in children typically resolve within several months and seem to be less common in young children. In our study the median age of children was 4 years and questionnaires were filled out starting at 6 months after acute infection. Therefore, the time points of 6 and 12 months used in this study might explain the low prevalence of persisting symptoms. Nonetheless, the measurable burden of long term symptoms may still be underreported in children. Potential reasons for this may be under recognition of signs and symptoms associated with long term symptoms and the fact that information was gathered using proxy reports which may be different from information obtained from patients directly.

Of note, those children who reported symptoms at 12 months after a household outbreak had not reported symptoms at 6 months follow-up, which suggests these might be new symptoms that developed during follow-up and do not represent persistent symptoms.

Limitations of this study were the relatively small sample size. Furthermore, it is unclear whether the presence of symptoms at 6 or 12 months follow up were persistent symptoms since initial diagnosis or have arisen during follow up due to other external factors. In addition, our study population consisted mostly of participants with a relatively high socioeconomic status and those who had few comorbidities. This might negatively impact the generalizability of our study findings.

Also, due to standardised RT-PCR test moments for all family members, a SARS-CoV-2 infection in children with a negative RT-PCR test cannot absolutely be excluded. In some cases the samples might have been collected to early or too late for viral detection. Furthermore, unfortunately no sufficient information about possible SARS-CoV-2 infections later on during the follow-up period was collected. Therefore, some long-term complaints reported by the control group might have been due to a recent SARS-CoV-2 or other viral infection.

Strengths of this study were the prospective set-up of the inclusion of a matched control group to distinguish the persistence of symptoms between participants with a SARS-CoV-2 infection and those with a SARS-CoV-2 negative RT-PCR test. In hindsight, the chosen time points for the questionnaires used to evaluate SARS-CoV-2 persisting symptoms was based on a lack of evidence regarding the course and possible duration of these symptoms. However, with the chosen time points in this study, we can verify the prevalence of persisting symptoms in children at 6 and 12 months after acute infection being scarce.

In conclusion, long-term symptoms after SARS-CoV-2 infection, or after acute respiratory illness from other viral causes within a SARS-CoV-2 negative household, were seldom reported at 6 months and 12 months in previously healthy children.

## Supplementary Information

**Additional file 1:** Pdf Questionnaire PedsQL 2-4 years.

**Additional file 2:** Pdf Questionnaire PedsQL 5-7 years.

**Additional file 3:** Pdf Questionnaire PedsQL 8-12 years.

**Additional file 4:** Pdf Questionnaire TAPQOL.

## Data Availability

The datasets used and/or analysed during the current study are available from the corresponding author on reasonable request.
