# Supplementary material for: Long term sequelae after SARS-CoV-2 infection in children: a household study
Source: Virol J. 2023 Jun 28;20:137. doi: 10.1186/s12985-023-02094-z (PMC10308779; doi:10.1186/s12985-023-02094-z)
Supplement: Supplementary file 1 — Additional file 1: Pdf Questionnaire PedsQL 2-4 years. [file 12985_2023_2094_MOESM1_ESM.pdf]

Ouders: PedsQL 2 t/m 4

voortgang lijst: 0 %

Kwaliteit van leven vragenlijst voor peuters (leeftijd 2-4 jaar) in te vullen door ouders

Hieronder staat een lijst van dingen die een probleem kunnen zijn voor uw kind. Kunt u ons vertellen **hoe vaak** uw kind in de **afgelopen week** met elk van deze dingen problemen heeft gehad? Vink het cijfer aan dat het beste van toepassing is. U kunt kiezen uit:

- **0** als het **nooit** een probleem is
- **1** als het **bijna nooit** een probleem is
- **2** als het **soms** een probleem is
- **3** als het **vaak** een probleem is
- **4** als het **bijna altijd** een probleem is

Er zijn geen goede of foute antwoorden. Als u een vraag niet begrijpt, vraag dan om hulp.

1
Lichamelijk functioneren
Hoe vaak heeft uw kind in de **afgelopen week** problemen gehad met:

	Nooit	Bijna nooit	Soms	Vaak	Bijna altijd
1. Lopen	0	1	2	3	4
2. Rennen	0	1	2	3	4
3. Actief spelen en lichaamsbeweging	0	1	2	3	4
4. Iets zwaars optillen	0	1	2	3	4
5. Baden	0	1	2	3	4
6. Helpen met opruimen van zijn/haar speelgoed	0	1	2	3	4
7. Pijn hebben	0	1	2	3	4
8. Weinig energie	0	1	2	3	4

2
Emotioneel functioneren
Hoe vaak heeft uw kind in de **afgelopen week** problemen gehad met:

	Nooit	Bijna nooit	Soms	Vaak	Bijna altijd
1. Zich angstig of bang voelen	0	1	2	3	4
2. Zich verdrietig of somber voelen	0	1	2	3	4
3. Zich boos voelen	0	1	2	3	4
4. Moeite met slapen	0	1	2	3	4
5. Zich zorgen maken/bang zijn	0	1	2	3	4

3

Sociaal functioneren

Hoe vaak heeft uw kind in de **afgelopen week** problemen gehad met:

	Nooit	Bijna nooit	Soms	Vaak	Bijna altijd
1. Spelen met andere kinderen	0	1	2	3	4
2. Andere kinderen willen zijn/haar vriend(in) niet zijn	0	1	2	3	4
3. Gepest worden door andere kinderen	0	1	2	3	4
4. Bepaalde dingen niet kunnen die andere kinderen van zijn/haar leeftijd wel kunnen	0	1	2	3	4
5. Mee kunnen blijven doen tijdens het spelen met andere kinderen	0	1	2	3	4

4

Gaat uw kind naar een kinderdagverblijf, een peuterspeelzaal of naar school?

- Nee
- Ja

PedsQL™ Copyright © 1998-2019 James W. Varni, Ph.D. All rights reserved. For any information on the use of the PedsQL™, please contact Mapi Research Trust, Lyon, France. Internet: https://eprovide.mapi-trust.org
