# Supplementary material for: Long term sequelae after SARS-CoV-2 infection in children: a household study
Source: Virol J. 2023 Jun 28;20:137. doi: 10.1186/s12985-023-02094-z (PMC10308779; doi:10.1186/s12985-023-02094-z)
Supplement: Supplementary file 2 — Additional file 2: Pdf Questionnaire PedsQL 5-7 years. [file 12985_2023_2094_MOESM2_ESM.pdf]

Ouders: PedsQL 5 t/m 7

VRAGENLIJST voor JONGE KINDEREN in te vullen door OUDERS (leeftijd 5-7)

TOELICHTING Op deze pagina staat een lijst van dingen die een probleem kunnen zijn voor uw kind.
Kunt u ons vertellen **hoe vaak** uw kind in de **afgelopen week** met elk van deze dingen problemen heeft gehad? Klik het bolletje aan bij:

- **0** als het **nooit** een probleem is,
- **1** als het **bijna nooit** een probleem is,
- **2** als het **soms** een probleem is,
- **3** als het **vaak** een probleem is,
- **4** als het **bijna altijd** een probleem is.

Er zijn geen goede of foute antwoorden. Als u een vraag niet begrijpt, vraag dan om hulp.

1
Lichamelijk functioneren
Hoe vaak heeft uw kind in de **afgelopen week** problemen gehad met...

	**Nooit**	**Bijna nooit**	**Soms**	**Vaak**	**Bijna altijd**
1. Meer dan één straat op en neer lopen	0	1	2	3	4
2. Rennen	0	1	2	3	4
3. Aan sport of andere lichaamsbeweging doen	0	1	2	3	4
4. Iets zwaars optillen	0	1	2	3	4
5. Zelfstandig een bad of douche nemen	0	1	2	3	4
6. Karweitjes doen, zoals het opruimen van zijn / haar speelgoed	0	1	2	3	4
7. Het hebben van wondjes of pijn	0	1	2	3	4
8. Weinig energie hebben	0	1	2	3	4

2
Emotioneel functioneren
Hoe vaak heeft uw kind in de **afgelopen week** problemen gehad met...

	**Nooit**	**Bijna nooit**	**Soms**	**Vaak**	**Bijna altijd**

1. Zich angstig of bang voelen	0	1	2	3	4
2. Zich verdrietig of somber voelen	0	1	2	3	4
3. Zich boos voelen	0	1	2	3	4
4. Moeite met slapen	0	1	2	3	4
5. Zorgen maken over wat hem/ haar zal overkomen	0	1	2	3	4

3

Sociaal Functioneren

Hoe vaak heeft uw kind in de **afgelopen week** problemen gehad met...

	**Nooit**	**Bijna nooit**	**Soms**	**Vaak**	**Bijna altijd**
1. Op kunnen schieten met andere kinderen	0	1	2	3	4
2. Andere kinderen die niet zijn/ haar vriend(in) willen zijn	0	1	2	3	4
3. Gepest worden door andere kinderen	0	1	2	3	4
4. Dingen niet kunnen die andere kinderen van zijn/ haar leeftijd wel kunnen	0	1	2	3	4
5. Mee kunnen blijven doen tijdens het spelen met andere kinderen	0	1	2	3	4

4

School functioneren

Hoe vaak heeft uw kind in de **afgelopen week** problemen gehad met...

	**Nooit**	**Bijna nooit**	**Soms**	**Vaak**	**Bijna altijd**
1. Opletten tijdens de les	0	1	2	3	4
2. Dingen vergeten	0	1	2	3	4
3. Bijblijven met schoolwerk	0	1	2	3	4
4. Niet naar school gaan omdat hij/zij zich niet lekker voelt	0	1	2	3	4
5. Niet naar school gaan omdat hij/zij naar de dokter of het ziekenhuis moet	0	1	2	3	4

PedsQL™ Copyright © 1998-2019 James W. Varni, Ph.D. All rights reserved. For any information on the use of the PedsQL™, please contact Mapi Research Trust, Lyon, France. Internet: https://eprovide.mapi-trust.org
