# Supplementary material for: Long term sequelae after SARS-CoV-2 infection in children: a household study
Source: Virol J. 2023 Jun 28;20:137. doi: 10.1186/s12985-023-02094-z (PMC10308779; doi:10.1186/s12985-023-02094-z)
Supplement: Supplementary file 3 — Additional file 3: Pdf Questionnaire PedsQL 8-12 years. [file 12985_2023_2094_MOESM3_ESM.pdf]

Ouders: PedsQL 8 t/m 12

VRAGENLIJST voor KINDEREN in te vullen door OUDERS (leeftijd 8-12)

TOELICHTING Op deze pagina staat een lijst van dingen die een probleem kunnen zijn voor uw kind.
Kunt u ons vertellen **hoe vaak** uw kind in de **afgelopen week** met elk van deze dingen problemen heeft gehad? Klik het bolletje aan bij:

0 als het **nooit** een probleem is
1 als het **bijna nooit** een probleem is
2 als het **soms** een probleem is
3 als het **vaak** een probleem is
4 als het **bijna altijd** een probleem is

Er zijn geen goede of foute antwoorden. Als u een vraag niet begrijpt, vraag dan om hulp.

1
Lichamelijk functioneren
Hoe vaak heeft uw kind in de **afgelopen week** problemen gehad met...

	**Nooit**	**Bijna nooit**	**Soms**	**Vaak**	**Bijna altijd**
1. Meer dan 100 meter lopen	0	1	2	3	4
2. Rennen	0	1	2	3	4
3. Deelnemen aan sportactiviteiten of lichamelijke oefeningen	0	1	2	3	4
4. Iets zwaars optillen	0	1	2	3	4
5. Zelfstandig een bad of douche nemen	0	1	2	3	4
6. Karweitjes in en om het huis doen	0	1	2	3	4
7. Pijn hebben	0	1	2	3	4
8. Zich moe voelen	0	1	2	3	4

2
Emotioneel functioneren
Hoe vaak heeft uw kind in de **afgelopen week** problemen gehad met...

	**Nooit**	**Bijna nooit**	**Soms**	**Vaak**	**Bijna altijd**

1. Zich angstig of bang voelen	0	1	2	3	4
2. Zich verdrietig of somber voelen	0	1	2	3	4
3. Zich boos voelen	0	1	2	3	4
4. Problemen met slapen	0	1	2	3	4
5. Zorgen maken over wat hem/ haar zal overkomen	0	1	2	3	4

3

Sociaal Functioneren

Hoe vaak heeft uw kind in de **afgelopen week** problemen gehad met...

	**Nooit**	**Bijna nooit**	**Soms**	**Vaak**	**Bijna altijd**
1. Op kunnen schieten met andere kinderen	0	1	2	3	4
2. Andere kinderen willen zijn/ haar vriend(in) niet zijn	0	1	2	3	4
3. Gepest worden door andere kinderen	0	1	2	3	4
4. Dingen niet kunnen die andere kinderen van zijn/ haar leeftijd wel kunnen	0	1	2	3	4
5. Mee kunnen blijven doen tijdens het spelen met andere kinderen	0	1	2	3	4

4

School functioneren

Hoe vaak heeft uw kind in de **afgelopen week** problemen gehad met...

	**Nooit**	**Bijna nooit**	**Soms**	**Vaak**	**Bijna altijd**
1. Opletten tijdens de les	0	1	2	3	4
2. Dingen vergeten	0	1	2	3	4
3. Bijblijven in de klas en met huiswerk	0	1	2	3	4
4. Niet naar school gaan omdat hij/zij zich niet lekker voelt	0	1	2	3	4
5. Niet naar school gaan omdat hij/zij naar de dokter of het ziekenhuis moet	0	1	2	3	4

PedsQL™ Copyright © 1998-2019 James W. Varni, Ph.D. All rights reserved. For any information on the use of the PedsQL™, please contact Mapi Research Trust, Lyon, France. Internet: https://eprovide.mapi-trust.org
