# Supplementary material for: Long term sequelae after SARS-CoV-2 infection in children: a household study
Source: Virol J. 2023 Jun 28;20:137. doi: 10.1186/s12985-023-02094-z (PMC10308779; doi:10.1186/s12985-023-02094-z)
Supplement: Supplementary file 4 — Additional file 4: Pdf Questionnaire TAPQOL. [file 12985_2023_2094_MOESM4_ESM.pdf]

Print

Nummer: [_ _ _ _ _ _ _ _ _]

TAPQOL

Vragenlijst

voor ouders van 1 t/m 5-jarige kinderen

Wilt u eerst onderstaande vragen beantwoorden?

Is het kind, waarover u deze lijst invult, een jongen of een meisje? ‰ jongen ‰ meisje

Wat is de geboortedatum van het kind? .... (maand) .... (jaar)

Wat is de datum waarop u deze vragenlijst invult? .... (dag) .... (maand) .... (jaar)

LUMC

TNO

© TNO Preventie en Gezondheid / LUMC (Nederland), 2001
NL38592.094.12–versie 1.0 22 maart 2021- CoKids follow-up study

INSTRUCTIE

Geachte mevrouw/mijnheer,

De vragen in deze lijst gaan over allerlei dingen die te maken hebben met de gezondheid van uw kind. U kunt de vragen beantwoorden door het antwoord aan te kruisen dat het beste bij uw kind past.

Bijvoorbeeld zo:

Had uw kind de afgelopen 3 maanden oorpijn?

Oorpijn ‰ nooit ‰ soms ‰ vaak

1

Mijn kind voelde zich daarbij:

Als uw kind nooit oorpijn had, zoals in het voorbeeld hierboven, kunt u door naar de volgende vraag.

Als uw kind ‘soms’ of ‘vaak’ oorpijn had, zet u een kruisje bij één van die antwoorden. Precies onder deze twee antwoorden vindt u de zin “Mijn kind voelde zich daarbij”. Daar geeft u vervolgens aan hoe uw kind zich daarbij voelde. Dus zo:

Had uw kind de afgelopen 3 maanden oorpijn?

Oorpijn ‰ nooit ‰ soms ‰ vaak

1

Mijn kind voelde zich daarbij:

‰ goed ‰ niet zo goed ‰ vrij slecht ‰ slecht

Daarna gaat u door naar de volgende vraag.

Dit was een voorbeeld.
Op de volgende bladzijde begint de vragenlijst.

© TNO Preventie en Gezondheid / LUMC (Nederland), 2001

Had uw kind in de afgelopen 3 maanden:

Maag- of buikpijn

1

‰ nooit ‰ soms ‰ vaak

Mijn kind voelde zich daarbij:

‰ goed ‰ niet zo goed ‰ vrij slecht ‰ slecht

Krampjes

2

‰ nooit ‰ soms ‰ vaak

Mijn kind voelde zich daarbij:

‰ goed ‰ niet zo goed ‰ vrij slecht ‰ slecht

Eczeem

3

‰ nooit ‰ soms ‰ vaak

Mijn kind voelde zich daarbij:

‰ goed ‰ niet zo goed ‰ vrij slecht ‰ slecht

Jeuk

4

‰ nooit ‰ soms ‰ vaak

Mijn kind voelde zich daarbij:

‰ goed ‰ niet zo goed ‰ vrij slecht ‰ slecht

Droge huid

5

‰ nooit ‰ soms ‰ vaak

Mijn kind voelde zich daarbij:

‰ goed ‰ niet zo goed ‰ vrij slecht ‰ slecht

Bronchitis

6

‰ nooit ‰ soms ‰ vaak

Mijn kind voelde zich daarbij:

‰ goed ‰ niet zo goed ‰ vrij slecht ‰ slecht

Moeite met de ademhaling of longproblemen

7

‰ nooit ‰ soms ‰ vaak

Mijn kind voelde zich daarbij:

‰ goed ‰ niet zo goed ‰ vrij slecht ‰ slecht

© TNO Preventie en Gezondheid / LUMC (Nederland), 2001

Was uw kind in de afgelopen 3 maanden:

Benauwd

8

‰ nooit ‰ soms ‰ vaak

Mijn kind voelde zich daarbij:

‰ goed ‰ niet zo goed ‰ vrij slecht ‰ slecht

Misselijk

9

‰ nooit ‰ soms ‰ vaak

Mijn kind voelde zich daarbij:

‰ goed ‰ niet zo goed ‰ vrij slecht ‰ slecht

© TNO Preventie en Gezondheid / LUMC (Nederland), 2001

Hoe sliep uw kind in de afgelopen 3 maanden?

Sliep uw kind onrustig?

10

‰ nooit ‰ soms ‰ vaak

Mijn kind voelde zich daarbij:

‰ goed ‰ niet zo goed ‰ vrij slecht ‰ slecht

Lag uw kind 's nachts wakker?

11

‰ nooit ‰ soms ‰ vaak

Mijn kind voelde zich daarbij:

‰ goed ‰ niet zo goed ‰ vrij slecht ‰ slecht

Huilde uw kind 's nachts?

12

‰ nooit ‰ soms ‰ vaak

Mijn kind voelde zich daarbij:

‰ goed ‰ niet zo goed ‰ vrij slecht ‰ slecht

Had uw kind moeite om de hele nacht door te slapen?

13

‰ nooit ‰ soms ‰ vaak

Mijn kind voelde zich daarbij:

‰ goed ‰ niet zo goed ‰ vrij slecht ‰ slecht

© TNO Preventie en Gezondheid / LUMC (Nederland), 2001

Hoe at en dronk uw kind in de afgelopen 3 maanden?

Had uw kind een slechte eetlust?

14

‰ nooit ‰ soms ‰ vaak

Mijn kind voelde zich daarbij:

‰ goed ‰ niet zo goed ‰ vrij slecht ‰ slecht

Had uw kind moeite om voldoende te eten?

15

‰ nooit ‰ soms ‰ vaak

Mijn kind voelde zich daarbij:

‰ goed ‰ niet zo goed ‰ vrij slecht ‰ slecht

Weigerde uw kind te eten?

16

‰ nooit ‰ soms ‰ vaak

Mijn kind voelde zich daarbij:

‰ goed ‰ niet zo goed ‰ vrij slecht ‰ slecht

© TNO Preventie en Gezondheid / LUMC (Nederland), 2001

Gedrag van uw kind in de afgelopen 3 maanden:

**Mijn kind was driftig**<br>17	‰ nooit	‰ soms	‰ vaak
**Mijn kind was agressief**<br>18	‰ nooit	‰ soms	‰ vaak
**Mijn kind was geprikkeld, geïrriteerd**<br>19	‰ nooit	‰ soms	‰ vaak
**Mijn kind was boos**<br>20	‰ nooit	‰ soms	‰ vaak
**Mijn kind was onrustig of ongeduldig tegen mij**<br>21	‰ nooit	‰ soms	‰ vaak
**Mijn kind was opstandig/dwars tegen mij**<br>22	‰ nooit	‰ soms	‰ vaak
**Mijn kind was voor mij onhandelbaar**<br>23	‰ nooit	‰ soms	‰ vaak

© TNO Preventie en Gezondheid / LUMC (Nederland), 2001

Hoe was uw kind in de afgelopen 3 maanden?

**Opgewekt** 24	‰ nooit	‰ soms	‰ vaak
**Blij** 25	‰ nooit	‰ soms	‰ vaak
**Gelukkig** 26	‰ nooit	‰ soms	‰ vaak
**Bang** 27	‰ nooit	‰ soms	‰ vaak
**Gespannen** 28	‰ nooit	‰ soms	‰ vaak
**Angstig** 29	‰ nooit	‰ soms	‰ vaak
**Energiek** 30	‰ nooit	‰ soms	‰ vaak
**Actief** 31	‰ nooit	‰ soms	‰ vaak
**Levendig** 32	‰ nooit	‰ soms	‰ vaak

© TNO Preventie en Gezondheid / LUMC (Nederland), 2001

Is uw kind *jonger* dan 1 jaar en 6 maanden, dan hoeft u de rest van deze vragenlijst **niet** in te vullen. Hartelijk bedankt voor uw medewerking!

Is uw kind *ouder* dan 1 jaar en 6 maanden,
dan kunt u gewoon doorgaan met het invullen van de vragen op de volgende bladzijden.

© TNO Preventie en Gezondheid / LUMC (Nederland), 2001

Gedrag van uw kind in de afgelopen 3 maanden, met andere kinderen:

Mijn kind kon met andere kinderen prettig spelen ‰ nooit ‰ soms ‰ vaak

33

Mijn kind was bij andere kinderen op zijn/haar gemak ‰ nooit ‰ soms ‰ vaak

34

Mijn kind was zeker van zichzelf bij andere kinderen ‰ nooit ‰ soms ‰ vaak

35

© TNO Preventie en Gezondheid / LUMC (Nederland), 2001

Had uw kind in de afgelopen 3 maanden <u>in vergelijking met</u> <u>leeftijdgenootjes</u>

Moeite met lopen?

36

‰ nee ‰ ja, een beetje ‰ ja, veel ‰ loopt (nog) niet

Mijn kind voelde zich daarbij:

‰ goed ‰ niet zo goed ‰ vrij slecht ‰ slecht

Moeite met rennen/hollen

37

‰ nee ‰ ja, een beetje ‰ ja, veel ‰ rent (nog) niet

Mijn kind voelde zich daarbij:

‰ goed ‰ niet zo goed ‰ vrij slecht ‰ slecht

Moeite om zonder hulp de trap op te lopen?

38

‰ nee ‰ ja, een beetje ‰ ja, veel ‰ loopt (nog) niet

Mijn kind voelde zich daarbij:

‰ goed ‰ niet zo goed ‰ vrij slecht ‰ slecht

Moeite met het bewaren van het evenwicht

39

‰ nee ‰ ja, een beetje ‰ ja, veel

Mijn kind voelde zich daarbij:

‰ goed ‰ niet zo goed ‰ vrij slecht ‰ slecht

© TNO Preventie en Gezondheid / LUMC (Nederland), 2001

Had uw kind in de afgelopen 3 maanden in vergelijking met leeftijdgenootjes:

Moeite om te begrijpen wat anderen zeiden?

40

‰ nooit ‰ soms ‰ vaak

Mijn kind voelde zich daarbij:

‰ goed ‰ niet zo goed ‰ vrij slecht ‰ slecht

Moeite om duidelijk te praten?

41

‰ nooit ‰ soms ‰ vaak

Mijn kind voelde zich daarbij:

‰ goed ‰ niet zo goed ‰ vrij slecht ‰ slecht

Moeite om uit zijn/haar woorden te komen?

42

‰ nooit ‰ soms ‰ vaak

Mijn kind voelde zich daarbij:

‰ goed ‰ niet zo goed ‰ vrij slecht ‰ slecht

Moeite om duidelijk te maken wat het wil?

43

‰ nooit ‰ soms ‰ vaak

Mijn kind voelde zich daarbij:

‰ goed ‰ niet zo goed ‰ vrij slecht ‰ slecht

Dit is het einde van de vragenlijst.
Hartelijk dank voor het invullen!

© TNO Preventie en Gezondheid / LUMC (Nederland), 2001
